# Administration of Albumin Solution Increases Serum Levels of Albumin in Patients With Chronic Liver Failure in a Single-Arm Feasibility Trial

**DOI:** 10.1016/j.cgh.2017.09.012

**Published:** 2018-05

**Authors:** Louise China, Simon S. Skene, Zainib Shabir, Alexander Maini, Yvonne Sylvestre, Kate Bennett, Scott Bevan, James O’Beirne, Ewan Forrest, Jim Portal, Steve Ryder, Gavin Wright, Derek W. Gilroy, Alastair O’Brien

**Affiliations:** ∗ Division of Medicine, University College London, United Kingdom; ‡ Comprehensive Clinical Trials Unit, University College London, United Kingdom; § Royal Free National Health Service Trust, London, United Kingdom; ‖ Glasgow Royal Infirmary, Glasgow, United Kingdom; ¶ Bristol Royal Infirmary, Bristol, United Kingdom; # Nottingham University Hospital, Nottingham, United Kingdom; ∗∗ Basildon University Hospital, Essex, United Kingdom

**Keywords:** Cirrhosis, Treatment, Mortality, Immune Response, ACLF, acute-on-chronic liver failure, AD, acute decompensation, ATTIRE, Albumin To PrevenT Infection In Chronic LiveR FailurE, HAS, human albumin solution, IDMC, Independent Data Monitoring Committee, PGE_2_, prostaglandin E_2_, RCT, randomized controlled trial, SAE, serious adverse event, TNF, tumor necrosis factor

## Abstract

**Background & Aims:**

Infections are life-threatening to patients with acute decompensation and acute-on-chronic liver failure (AD/ACLF). Patients with AD/ACLF have prostaglandin E2–mediated immune suppression, which can be reversed by administration of albumin; infusion of 20% human albumin solution (HAS) might improve outcomes of infections. We performed a feasibility study to determine optimal trial design, assess safety, and validate laboratory assessments of immune function to inform design of a phase 3 trial.

**Methods:**

We performed a prospective multicenter, single-arm, open-label trial of 79 patients with AD/ACLF and levels of albumin lower than 30 g/L, seen at 10 hospitals in the United Kingdom from May through December 2015. Patients were given daily infusions of 20% HAS, based on serum levels, for 14 days or until discharge from the hospital. Rates of infection, organ dysfunction, and in-hospital mortality were recorded. The primary end point was daily serum albumin level during the treatment period. Success would be demonstrated if 60% achieved and maintained serum albumin levels at or above 30 g/L on at least one third of days with recorded levels.

**Results:**

The patients’ mean model for end-stage disease score was 20.9 ± 6.6. The primary end point (albumin ≥30 g/L on at least one third of days recorded) was achieved by 68 of the 79 patients; 75% of administrations were in accordance with suggested dosing regimen. Mean treatment duration was 10.3 days (104 ± 678 mL administered). There were 8 deaths and 13 serious adverse events, considered by the independent data-monitoring committee to be consistent with those expected. Twelve of 13 patients that developed either respiratory or cardiovascular dysfunction (based on ward-based clinical definitions) as their only organ dysfunction were alive at 30 days compared with 1 of 3 that developed renal dysfunction. Only 1 case of brain dysfunction was recorded.

**Conclusions:**

In a feasibility trial, we found that administration of HAS increased serum levels of albumin in patients with AD/ACLF. The dosing regimen was acceptable at multiple sites and deemed safe by an independent data-monitoring committee. We also developed a robust system to record infections. The poor prognosis for patients with renal dysfunction was confirmed. However, patients with cardiovascular or respiratory dysfunction had good outcomes, which is counterintuitive. Severe encephalopathy appeared substantially under-reported, indicating that ward-based assessment of these parameters cannot be recorded with sufficient accuracy for use as a primary outcome in phase 3 trials. Trial registration no: EudraCT 2014-002300-24 and ISRCTN14174793.

See editorial on page 633, and related article on page 738.

Liver disease represents 2% to 3% of deaths globally^1^, ^2^ and was estimated to be responsible for more than 1 million deaths in 2010.^1^ Incidence rates are predicted to double over the next 20 years.^3^

Cirrhosis patients with liver failure are termed acute decompensation (AD) or acute-on-chronic-liver failure^4^ (ACLF). They are highly prone to bacterial infection^5^, ^6^ secondary to immune dysfunction,^7^ with nosocomial infection rates of 35% compared with 5% in noncirrhotic patients.^8^ Bacterial infection with organ dysfunction carries a mortality risk ranging from 60% to 95%.^9^, ^10^ Currently, there is no immune-restorative treatment for patients with AD/ACLF.

Intravenous albumin is used commonly in patients with AD/ACLF as a volume expander^11^, ^12^, ^13^ and has been proven to prevent and improve renal failure.^14^, ^15^ Many hepatologists believe it has additional properties,^16^ however, no multicenter evidence exists for these putative properties and critical-care studies have failed to establish a role.^17^

In our previous work, we showed that increased circulating prostaglandin E_2_ (PGE_2_) was a potential key immune-suppressive mediator in AD.^18^ We also developed an immune assay to assess the effects of patient plasma on healthy monocyte-derived macrophage tumor necrosis factor (TNF) production because monocyte deactivation is a key feature of AD/ACLF and reduced monocyte TNF production predicts poor outcomes in sepsis.^11^ We recently showed, using a whole-blood stimulation assay, a reduction in TNF production by fresh circulating monocytes taken from ACLF patients. These data match our observations made when monocyte-derived macrophages are treated with AD/ACLF plasma, validating our approach.^12^ The only clinical characteristic that predicted immune suppression using this approach was a serum albumin value less than 30 g/L. Albumin has been reported to bind and catalyze inactivation of PGE_2_,^13^ and both circulating levels and binding capacity^19^ decrease in AD/ACLF, making PGE_2_ potentially more bioavailable. In our pilot study, ex vivo immune function improved after 20% human albumin solution (HAS) transfusions.^18^ Therefore, we hypothesized that infusing albumin in hospitalized AD/ACLF patients with a serum albumin value less than 30 g/L with a target to increase this value to 35 g/L or higher would improve immune function, prevent new infections, and thus improve mortality. In view of the current discrepancies in ACLF definition^20^ we believed that large-scale recruitment required simple inclusion criteria and wished to test whether basing these on a serum albumin level less than 30 g/L would select appropriate patients.

There were several other uncertainties regarding our protocol that required clarification before embarking on a large, randomized controlled trial (RCT) comparing albumin infusions with standard care. These included the use of a 72-hour window from admission to treatment, our end point definitions, and obtaining daily data and sample collection. Furthermore, only 1 previous UK multicenter interventional AD/ACLF trial has been performed, the Steroids or Pentoxifylline for Alcoholic Hepatitis trial.^21^ Furthermore, a recent HAS infusion in sepsis study did not achieve the 30 g/L target^22^ and a HAS in cirrhosis trial was stopped early because of potential adverse effects. Therefore, a feasibility study was essential to determine whether our suggested protocol was achievable, effective, and did not suggest safety concerns. We did not include a standard-care arm in this feasibility trial and the study was not powered to detect clinical outcomes.

## Materials and Methods

### Study Design

We conducted this prospective, multicenter, open-label, feasibility trial of targeted 20% HAS infusions in UK AD/ACLF patients to inform the design of a phase 3 RCT. A protocol report was published^23^ and the full protocol is available online. All authors had access to the study data, and reviewed and approved the manuscript.

### Inclusion Criteria

All patients admitted to the hospital with AD or severe worsening of the complications of cirrhosis, who were older than age 18 years, with a serum albumin level less than 30 g/L, a predicted hospital admission longer than 5 days, and that were for full active management at admission were eligible. Patients were recruited within 72 hours of admission; the full inclusion criteria are listed in Supplementary Table 1. We sought written informed consent from patients or their representatives if patients were lacking decision-making capacity. Research ethical approval was granted by London-Brent research ethics committee (ref: 15/LO/0104).

### Intervention

In-patients received daily 20% HAS infusions intravenously for the treatment period, with a maximum of 14 days or until the patient was considered medically fit for discharge if fewer than 14 days. The volume of HAS prescribed was determined by the patients’ serum albumin level that day (Supplementary Table 2), based on the Albumin Replacement in Patients with Severe Sepsis study^22^ and clinical experience. All clinicians could deviate from the suggested regimen, but were requested to document their reasons for doing so.

### Clinical Outcomes

The primary end point was daily serum albumin value for the treatment period. Success was defined as 60% of patients achieving and maintaining serum albumin levels at or greater than 30 g/L on at least one third of the days in the study with recorded levels.

The rates of infection, organ dysfunction (for definitions see Supplementary Table 3, Supplementary Table 4), and in-hospital mortality, the component elements of the planned composite end point for the RCT, also were recorded. Definitions of organ failure were chosen from the European Foundation for the study of chronic liver failure Sequential Organ Failure Assessment score.^24^ We used a recent definition of renal dysfunction in cirrhosis^25^ reflecting deterioration in patient renal function rather than static values. Because studies have shown that up to 50% of liver patients considered to be infected may have negative microbiological culture,^26^ we used antibiotic initiation as a surrogate for infection diagnosis. A subset of patients had clinical, biochemical, and microbiological data recorded for validation of infection diagnosis.

Data were summarized further within groups defined by baseline serum albumin levels (<20, 20–25, and 26–29 g/L) to investigate any apparent differences in outcome by group. This subgroup analysis was considered crucial to potentially identify whether any protocol amendments were necessary. The information was summarized for the total volume of albumin infused and of fluid administered, days spent in the intensive care unit, and the duration of hospital stay. Safety was assessed by our Independent Data Monitoring Committee (IDMC) who considered the number of serious adverse events (SAEs) reported during the trial.

### Independent Data Monitoring Committee

The IDMC received monthly updates of the SAEs to ensure robust monitoring and met every 6 months to report to the Trial Steering Committee. The SAEs were recorded from entry into study until discharge or the end of treatment.

### Statistical Methods

A sample size of 80 ensured 72 evaluable patients (this assumed a 10% loss to follow-up evaluation/withdrawal), with a probability of achieving 44 or more successes of 80%. Calculations assumed each patient had a 65% chance of albumin level being 30 g/L or greater on at least one third of the days when the level was recorded. If 44 successes or more were observed, then a single-sided 90% CI would suggest that the true rate is higher than 50%. Because this was a feasibility study, the emphasis was on producing relevant data summaries rather than on formal modeling or hypothesis testing. All analyses were undertaken by an intention-to-treat, including all consented and enrolled patients for whom outcomes were available, and according to a prespecified (approved before database lock) statistical analysis plan that is included in the Supplementary Materials and Methods section.

### Trial Registration

The trial is registered with the European Medicines Agency (European Clinical Trials Database 2014-002300-24) and adopted by National Institute for Health Research (International Standard Randomised Controlled Trial Number 14174793).

## Results

### Recruitment and Baseline Clinical Characteristics

**Figure 1:**
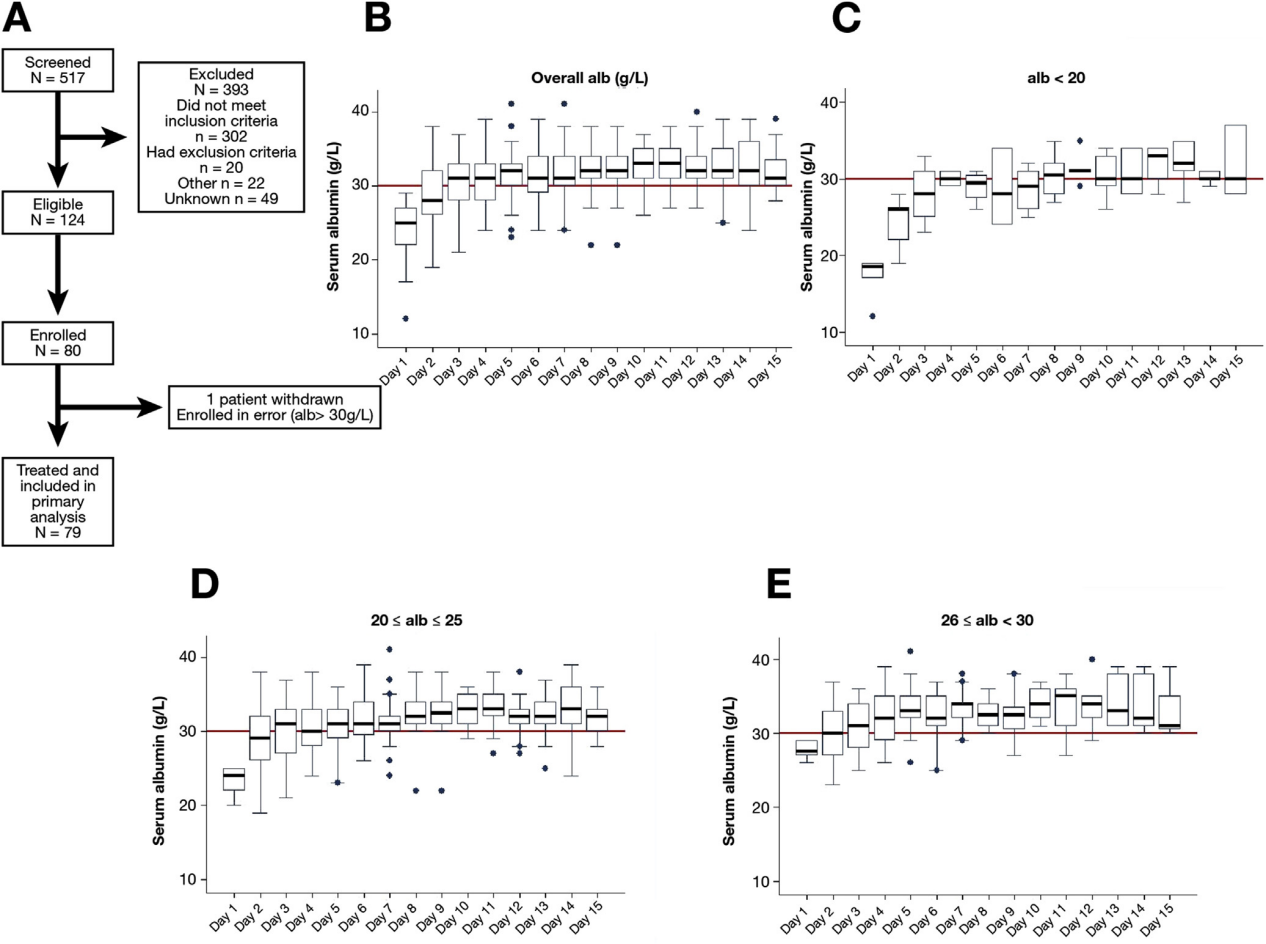
Targeted 20% HAS infusions according to the Albumin To PrevenT Infection In Chronic LiveR FailurE protocol effectively increase serum albumin to 30 g/L or greater. (*A*) Albumin To PrevenT Infection In Chronic LiveR FailurE feasibility study Consolidated Standards of Reporting Trials flowchart. (*B*) Median serum albumin levels throughout the study period. (*C–E*) Data are expressed according to baseline serum albumin (alb) level. Day 1 was defined as the time of recruitment (pretreatment). The *horizontal line* in the *boxes* indicates the median, the *top* and *bottom* of the *box* indicate the interquartile range; *dots* represent individual outliers, defined as data points greater than 1.5 times the interquartile range from the median.

Between May and December 2015, there were 517 AD/ACLF patients who were screened at 10 UK hospitals, with 124 eligible for inclusion and 80 enrolled. One patient was excluded from analysis (Figure 1*A*) because their baseline albumin level was greater than 30 g/L. Their mean age was 53.4 years, with a male predominance (66%), and alcohol was the primary underlying cause in 96% (Table 1). The mean Model for End-stage Liver Disease score was 20.9 (SD, 6.62), 17 of 79 patients had 1 or more extrahepatic organ dysfunction(s) at baseline according to our definitions, and 21 patients had ACLF grades 1 to 3 according to the European Association for the Study of the Liver-CLIF definition.^27^ Sixty-seven percent of patients had baseline albumin levels less than 25 g/L. The mean time from admission to enrollment was 1.8 days. The most common reasons for ineligibility during screening were as follows: (1) albumin level of 30 g/L or greater; (2) admission more than 72 hours before screening; and (3) predicted hospital stay of fewer than 5 days. The most common reasons for nonparticipation of eligible patients were as follows: (1) patient declined; (2) informed consent was not possible; and (3) the site was unable to randomize within 72 hours.

**Table 1:** Baseline Demographics and Clinical Characteristics of the Analysis Population (n = 79)

Characteristic	Mean (SD)
Age, *y*	53.41 (11.63)
Serum albumin level, *g/L*	23.95 (3.51)
Days since admission	1.81 (0.88)
MELD	20.90 (6.62)
Creatinine level	91.2 (78.2)
	n (%)
Male	52 (66)
Admitted to ICU	2 (3)
Prescribed antibiotics	41 (52)
Diagnosis of infection	27 (34)
Etiology of cirrhosis^a^	
Alcohol	76 (96)
Hepatitis B	1 (1)
Hepatitis C	11 (14)
NAFLD	4 (5)
Other etiologies	2 (3)
Organ dysfunction^b^	
Renal	8 (10)
Respiratory	9 (11)
Circulatory	13 (16)
Cerebral	3 (4)
ACLF grade^c^	
Grade 0	58 (73)
Grade 1	11 (14)
Grade 2	6 (8)
Grade 3	4 (5)

ACLF, acute-on-chronic liver failure; ICU, intensive care unit; MELD, Model for End-stage Liver Disease; NAFLD, nonalcoholic fatty liver disease.

a Some patients have more than 1 cirrhosis etiology.

b Organ dysfunction at baseline was determined according to the following criteria: renal, creatinine level > 133 μmol/L; respiratory, oxygen saturations divided by inspired oxygen < 357; circulatory, mean arterial pressure < 60 mm Hg or patient is receiving inotropic/vasopressor support; cerebral, grade III or IV encephalopathy.

c According to European Association for the Study of the Liver-CLIF criteria.

### Targeted 20% Human Albumin Solution Infusions Increase Serum Albumin Level to 30 g/L or More

A total of 68 of 79 patients achieved a serum albumin level of 30 g/L or greater on at least one third of days recorded, providing a success rate of 86% (95% CI, 76%–92%) (Table 2, and Supplementary Table 5). All patients enrolled contributed to the analysis. The regimen was effective across all serum albumin subgroups, with the highest success in the 26 to 29 g/L group (96% success; 95% CI, 80%–100%) compared with less than 20 g/L (50% success; 95% CI, 19%–81%). The mean treatment length was 10.3 days and a mean of 1042 mL 20% HAS (SD, 678 mL) was administered; more than 50% of patients had their albumin level restored to 30 g/L or greater after 3 days, and more than 75% after 7 days. Deaths (n = 8) or SAEs (n = 12) during the trial period were not considered directly related to the HAS infusions by the IDMC (Table 3). Sixty-four percent of administrations were in accordance with the suggested protocol, with 88% within ±100 mL of the suggested dose (Supplementary Table 6). No patient underwent a liver transplantation. On 161 of 657 occasions, 20% HAS was not administered despite a serum albumin level less than 35 g/L, suggesting an adherence rate of 75%.

**Table 2:** Primary Outcome of Daily Albumin Level for Subgroups Defined by Baseline Albumin Level (<20, 20–25, and 26–29 g/L)

Baseline albumin level, *g/L*	Successes	Estimated success probability (%), mean (95% CI)
<20	5/10	50 (19–81)
20–25	38/43	88 (75–96)
26–29	25/26	96 (80–100)

NOTE. The following successes were observed in the patient population.

**Table 3:** Details of Reported Serious Adverse Events Throughout Trial Treatment Period (Days 1–15)

SAE description	Events, n
New ascites	1
Renal impairment	1
Variceal bleeding (death)	3
Variceal bleeding (death)	1
Pneumonia (death)	1
Death (decompensated cirrhosis)	4
Bronchogenic carcinoma and pleural effusion (death)	1
Total deaths in trial treatment period	8 (10%)

SAE, serious adverse event.

### Infection, Organ Dysfunction, Death, and Duration of Hospital Stay

Clinical end points were reported from days 3 to 15 after study entry (termed the *treatment period*) to ensure at least 24 hours since the patients first received 20% HAS treatment before measuring possible outcomes. The average time from admission to death or discharge was 16.9 days (SD, 15.7 d; range, 2–102 d). Forty-two of 817 patient days recorded were in the intensive care unit (approximately 5%). Supplementary Table 7 shows the data for days 2 to 15.

#### Infection defined by new/change in antibiotic prescription

Forty-one of 79 patients were prescribed antibiotics at recruitment to the study, with 27 of 41 given an infection diagnosis by their clinician. During the treatment period, 21 of 79 patients (27%) were diagnosed with a new infection (Table 4). The patients who had been prescribed antibiotics on admission had increased subsequent nosocomial infection rates compared with those who were not prescribed antibiotics on admission (24% vs 8%, respectively).

**Table 4:** Number of Patients Reported as Having Infection, Organ Dysfunction, and Death During the Trial Treatment Period

End point	Patients, n, days 3–15 of trial treatment
Infection	21/79 (27%)
Extrahepatic organ dysfunction	
Renal	7/79 (9%)
Respiratory	19/79 (24%)
Circulatory	15/79 (19%)
Cerebral	1/79 (1%)
Death	5/79 (6%)

NOTE. Data were recorded throughout the study period, but data were reported from day 3 of treatment onward so that patients had at least 24 hours of intravenous 20% HAS treatment. Patients may have achieved more than 1 end point (eg, infection and death). Organ dysfunction at baseline was determined according to the following criteria: renal, creatinine level > 133 μmol/L; respiratory, oxygen saturations divided by inspired oxygen < 357; circulatory, mean arterial pressure < 60 mm Hg or patient is receiving inotropic/vasopressor support; cerebral, grade III or IV encephalopathy.

Infection case report forms were completed for 35 of the antibiotic prescriptions (either at baseline or after recruitment). A blinded microbiology review showed that 4 of 35 did not fulfill infection predefined criteria (Supplementary Table 4). Supplementary Table 8 details these case report form data.

#### Extrahepatic organ dysfunction

Overall, 7 of 79 (9%) patients met renal dysfunction criteria, 19 of 79 (24%) met respiratory dysfunction criteria, 15 of 79 (19%) met circulatory dysfunction criteria, and 1 of 79 (1%) met cerebral dysfunction criteria during the treatment period.

#### Death

Five patients (6%) died during the treatment period, and 14 patients died within 30 days of study entry (18%).

#### The planned composite end point for the randomized controlled trial

Thirty-eight of 79 patients (48%) reached the planned composite end point during the treatment period (Table 5). Of these, the breakdown of components that triggered the composite end point first were as follows.

1. Thirteen patients had an infection recorded as the first end point component, with 8 patients developing subsequent organ dysfunction and 4 of these patients had died by 30 days. Of the 5 patients who did not develop subsequent organ dysfunction, 1 patient died within 30 days. The median hospital stay was 19 days after infection diagnosis (Supplementary Table 9).
2. Three patients developed renal dysfunction as the first end point, of these, 2 patients died during admission and the other patient was alive at 30 days. A further 4 patients developed renal dysfunction after another organ dysfunction, with 2 patients dying as inpatients (1 patient during the treatment period), another patient shortly after discharge, and the other patient was alive at 30 days (Supplementary Table 9).
3. Twelve patients developed respiratory dysfunction as the first end point, with 5 of these patients triggering subsequent other end points (4 infections, 1 died). Of the 7 patients who developed solely respiratory dysfunction, the vast majority had a good outcome, with 6 alive at 30 days, 1 death during admission, and a median stay from respiratory dysfunction diagnosis of only 5 days, with 3 patients discharged within 3 days of diagnosis with respiratory dysfunction (Supplementary Table 9).
4. Nine patients developed circulatory dysfunction as the first end point, with 3 patients triggering other end points, and 1 patient died during admission. All 6 patients who solely developed circulatory dysfunction were alive at 30 days, and the median stay after circulatory dysfunction diagnosis was 7 days, with 2 patients discharged within 2 days of diagnosis (Supplementary Table 9).
5. One patient died without previously triggering another end point.

**Table 5:** Incidence of Planned and Revised RCT Composite End Point and Contributing Components (Days 3–15 of Trial Treatment)

Patients (N = 79)	Planned RCT composite end point	Revised RCT composite end point, excluding respiratory, circulatory, and cerebral dysfunction
Composite end point	38 (48%)	25 (32%)
Infection^a^	13	19
Extrahepatic organ dysfunction^b^		
Renal	3	4
Respiratory	12	
Circulatory	9	
Cerebral	0	
Death	1	2

RCT, randomized controlled trial.

a Infection indicated by new or change in prescription for antibiotics.

b Organ dysfunction at baseline was determined according to the following criteria: renal, creatinine level > 133 μmol/L; respiratory, oxygen saturations divided by inspired oxygen < 357; circulatory, mean arterial pressure < 60 mm Hg or patient is receiving inotropic/vasopressor support; cerebral, grade III or IV encephalopathy.

Only 1 patient developed grade 3 hepatic encephalopathy after an infection, therefore no brain dysfunction diagnoses contributed to the end point.

### Serious Adverse Events

The SAEs (n = 13) during treatment were deemed to be unrelated to HAS by our IDMC (Supplementary Table 10).

## Discussion

Targeted albumin infusions effectively increased serum albumin levels to 30 g/L or greater in hospitalized AD/ACLF patients; a level below which we previously identified as predicting immune dysfunction.^18^ Our infusion protocol was effective, acceptable at multiple sites, and deemed safe by the IDMC.

Our suggested protocol allowed dose reduction (eg, if safety concerns), as clinical practice remains variable.^28^ We achieved the primary end point with 75% adherence, increasing and maintaining albumin even in patients with very low baseline values. Although a single-arm study, the rates of adverse events of particular concern were low, in particular there were no reports of pulmonary edema. Four variceal bleeds were reported, which potentially can be precipitated by increased portal pressure after albumin. Although not reported in previous albumin trials, this remains a concern. However, a 5% incidence during treatment is similar to expected rates.^21^, ^24^, ^29^ The IDMC had no reason to alter our regimen; the absolute safety of our albumin regimen will be determined in our RCT vs standard care.

Our inclusion criteria were broad and straightforward and selected patients with almost exclusively alcohol-induced liver disease, a substantial spread of albumin values, and an ACLF score of 1 to 3 in 25% of cases. We reasoned that our intervention would be more successful in ward-based patients rather than in patients with multi-organ failure, and these criteria appeared to capture exactly this population. The majority were recruited within 2 days of admission, which is crucial because early intervention is widely believed to be imperative to improve outcomes in critically ill patients. As a trial aimed at the prevention of nosocomial infection, the primary composite end point will be reported from day 3 onward in line with established diagnostic criteria, which also ensures at least 24 hours of 20% HAS therapy. The observed in-hospital and 30-day mortality were similar to previous studies.^21^, ^30^, ^31^ Survival at 3 and 6 months were not recorded but will be reported in the RCT. We included a medically fit for discharge category at the end of the trial period because many patients have social needs that prolong hospital stay beyond their medical needs.

Our RCT primary composite end point was planned as infection, organ dysfunction, and death because infection commonly triggers organ dysfunction and the combination substantially increases mortality. However, the feasibility of recording such data in a ward-based trial of AD/ACLF at multiple sites is not commonplace. Other than renal failure,^32^ there is also no universally accepted definition for early (reversible) organ dysfunction/failures in patients with cirrhosis.

A robust diagnosis of infection in AD/ACLF is challenging because of the high rates of culture-negative sepsis.^26^ The on-site clinician-reported infection rate on admission was 34%, which is in line with other studies; however, antibiotics were prescribed in substantially more patients (52%). This perhaps reflects a tendency to overprescribe, as reported elsewhere,^33^, ^34^ and therefore using a new/changed antibiotic prescription as a surrogate for infection diagnosis, as originally intended, appeared subject to potential bias and this cannot be standardized across multiple sites. In the RCT we therefore will define infection according to clinician diagnosis, triggering completion of an infection case report form, with a substantial proportion blindly scrutinized by a microbiology panel. This approach appeared feasible in a subset of 35 patients tested. Our data suggest that patients prescribed antibiotics at admission have a 3 times increased risk of subsequent nosocomial infection. This has been reported previously^6^ and justifies inclusion of these patients in an RCT to prevent infection; furthermore, an antibiotic prescription will be included in stratification at randomization. Whether our reported nosocomial infection rate of 27% represents a beneficial effect of 20% HAS infusions will be determined in the RCT.

Renal dysfunction uses an objective measurement, creatinine, and patients developing this had a poor prognosis, as expected. Renal dysfunction rates were lower than anticipated (9% vs up to ≈60%^35^), perhaps related to additional fluid.^30^ Cerebral, respiratory, and cardiac dysfunctions were recorded daily. Only 1 patient developed cerebral dysfunction reflecting severe hepatic encephalopathy (≥grade 3), suggesting under-reporting, and objective assessment of encephalopathy is recognized as challenging.^34^, ^36^ The majority of patients who solely triggered respiratory and cardiovascular end points had a good outcome, with several discharged within a few days. This is counterintuitive because organ dysfunction is a key predictor of poor prognosis. Assessment may be subject to technical difficulties such as a standard-size blood pressure cuff for patients with sarcopenia and a oxygen saturation/fraction of inspired oxygen recording of respiratory dysfunction is influenced greatly by the amount of oxygen delivered by mask or nasal cannulae. We believe our data cast significant doubt over whether these dysfunctions can be recorded accurately in largely ward-based patients across multiple sites and therefore precludes use as part of our RCT primary composite end point; although these will be reported. Table 5 shows the incidence of our revised primary composite end point of infection, renal dysfunction, and mortality.

In summary, our multicenter feasibility study represents a highly pragmatic feasibility study in a challenging group of patients. The data presented confirm that our suggested protocol was deliverable across multiple sites and the dosing regimen increased serum albumin without significant safety concern. Albumin To PrevenT Infection In Chronic LiveR FailurE stage 2 is a multicenter, open-label, interventional RCT and began recruitment in April 2016. This is a phase 3 RCT to verify whether targeting a serum albumin level of 35 g/L or greater in patients admitted with acutely decompensated cirrhosis using repeated intravenous infusions of 20% HAS will reduce the incidence of infection, renal dysfunction, and mortality for the treatment period (maximum 14 days, or discharge if <14 days) compared with standard medical care. The trial will recruit 866 patients at more than 30 sites across the United Kingdom.

Large multicenter studies in patients with advanced liver disease rarely are performed and require significant resources. This feasibility study has enabled substantial improvement in design and end point definition/selection as well as providing important safety information and mechanistic insight. This approach could serve as a model for future trials in such patients.

## Figures and Tables

**Supplementary Table 1:** ATTIRE Feasibility Study Patient Inclusion and Exclusion Criteria

Inclusion criteria	Exclusion criteria
All patients admitted to the hospital with acute onset or worsening of complications of cirrhosis	Advanced hepatocellular carcinoma with life expectancy of <8 weeks
Older than 18 years of age	Patients who will receive palliative treatment only during their hospital admission
Predicted hospital admission >5 days at trial enrollment, which must be within 72 hours of admission	Patients who are pregnant
Serum albumin level <30 g/L at screening	Known or suspected severe cardiac dysfunction
Documented informed consent to participate (or consent given by a legal representative)	Any clinical condition that the investigator considers would make the patient unsuitable for the trial
	The patient has been involved in a clinical trial of Investigational Medicinal Products within the previous 30 days that would impact their participation in this study
	Trial investigators unable to identify the patient (by NHS number)

ATTIRE, Albumin To PrevenT Infection In Chronic LiveR FailurE; NHS, National Health Service.

**Supplementary Table 2:** Suggested Daily Dosing Protocol for 20% HAS Administration According to Measured Serum Albumin Level

Patient serum albumin level, *g/L*	Suggested volume of 20% HAS to be infused at rate of 100 mL/h
≥35	None
30–34	100 mL
26–29	200 mL
20–25	300 mL
<20	400 mL

**Supplementary Table 3:** Definitions of Extrahepatic Organ Dysfunction

Organ	Definition of new dysfunction
Renal	Serum creatinine level increases by ≥50% as compared with serum creatinine level at baseline
Respiratory	Any single point increase in oxygen saturations divided by inspired oxygen as classified on the following scoring system compared with oxygen saturations divided by inspired oxygen at randomization: 0, >357; 1, >214 to ≤357; 2, ≤214 or mechanical ventilation
Circulatory	MAP decreases to <60 mm Hg, OR patient is started on inotropic/vasopressor support If the patient has MAP < 60 mm Hg at baseline, they will need to be started on inotropic/vasopressor support to reach this end point
Cerebral	Grade III (drowsy) or grade IV encephalopathy (coma) using the Westhaven criteria to grade hepatic encephalopathy If the patient has grade III encephalopathy (somnolent but rousable at baseline), they will need to progress to grade IV to reach this end point

NOTE. Organ dysfunction at baseline was determined according to the following criteria: renal, creatinine level > 133 μmol/L; respiratory, oxygen saturations divided by inspired oxygen < 357; circulatory, MAP < 60 mm Hg or patient is receiving inotropic/vasopressor support; cerebral, grade III or IV encephalopathy.

MAP, mean arterial pressure.

**Supplementary Table 4:** Definition of Infections–Infection Can Be Defined According to the Following Peer-reviewed Criteria^6^, ^14^

Type of infection	Definition
Spontaneous bacteremia	Positive blood cultures without a source of infection
SBP	Ascitic fluid polymorphonuclear cells >250 cells/mm^3^
Lower respiratory tract infections	New pulmonary infiltrates in the presence of: at least 1 respiratory symptom (cough, sputum production, dyspnea, pleuritic pain), with at least 1 finding on auscultation (rales or crepitation) or 1 sign of infection (core body temperature >38°C or <36°C, shivering, or leukocyte count >10,000/mm^3^ or <4000/mm^3^) in the absence of antibiotics
*Clostridium difficile* infection	Diarrhea with a positive *C difficile* assay
Bacterial enterocolitis	Diarrhea or dysentery with a positive stool culture for *Salmonella*, *Shigella*, *Yersinia*, *Campylobacter*, or pathogenic *Escherichia coli*
Soft-tissue/skin infection	Fever with cellulitis
Urinary tract infection	Urine white blood cell count >15/high-power field with either positive urine Gram stain or culture
Intra-abdominal infections	Diverticulitis, appendicitis, cholangitis, and so forth
Other infections	Not covered in 1–8
Fungal infections	
Nosocomial infections	Are diagnosed after 48 hours of admission, whereas secondary infections are diagnosed after a separate first infection has been documented

SBP, spontaneous bacterial peritonitis.

**Supplementary Table 5:** Daily Albumin Level (g/L) From Day 1 (Baseline) to Day 15: All Patients

Albumin level, *g/L*	Day														
	1	2	3	4	5	6	7	8	9	10	11	12	13	14	15
Median	25	28	31	31	32	31	31	32	32	33	33	32	32	32	31
IQR	22–27	26–32	28–33	28–33	30–33	29–34	30–34	31–34	31–34	31–35	31–35	31–34	31–35	30–36	30–34
Range	12–29	19–38	21–37	24–39	23–41	24–39	24–41	22–38	22–38	26–37	27–38	27–40	25–39	24–39	28–39
Mean (SD)	24 (4)	28 (4)	30 (4)	31 (3)	32 (3)	31 (3)	32 (3)	32 (3)	32 (3)	33 (3)	33 (3)	32 (3)	33 (3)	33 (3)	32 (3)
N	79	74	63	57	53	51	51	51	41	34	35	31	32	27	20
≥30, N	0	30	35	36	40	38	40	47	38	30	31	25	29	25	18
Study, N	79	77	74	72	65	60	57	55	48	44	43	42	39	34	28

IQR, interquartile range.

**Supplementary Table 6:** Number of Occasions Albumin Was Neither Prescribed nor Administered When Albumin Level <35 g/L

Days	1	2	3	4	5	6	7	8	9	10	11	12	13	14	Total
Neither prescribed nor administered	1	5	10	10	11	7	9	4	9	10	13	11	7	7	114
Prescribed but not administered	10	6	8	2	1	2	2	3	2	4	2	1	3	1	47
Administered but not prescribed	2	2	1	1	3	0	0	0	0	2	0	2	1	0	14
Total	13	13	19	13	15	9	11	7	11	16	15	14	11	8	175
Albumin level <35, N	79	73	64	58	56	48	48	44	40	32	29	34	28	24	657

**Supplementary Table 7:** Incidence of Proposed Composite End Point and Contributing Components, With and Without Respiratory/Circulatory Dysfunctions From Days 2–15

Patients (N = 79)	Existing composite end point, days 2–15, n (%)	Excluding respiratory, circulatory, and brain dysfunction from the composite end point, days 2–15, n (%)
Composite end point	44 (56)	30 (38)
Infection	10	21
Renal	4	5
Respiratory	17	
Circulatory	10	
Brain	0	
Death	3	4

**Supplementary Table 8:** Details From Infection Data Matched to 35 of 62 Antibiotic Prescriptions

Classified infection	Times confirmed, n^a^	Antibiotic sensitivity		
		Resistant	Sensitive^b^	Unknown
Spontaneous bacterial peritonitis	4	0	1	3
Pneumonia	6	0	1	5
Cellulitis	4	0	1	1
Bacterial enterocolitis	1	1^c^	0	0
Fungal infection	1	0	0	1
Spontaneous bacteremia	7	2^d^	2	3
Other infection	8	3^e^	0	5
Urinary tract infection	0	N/A		
Other intra-abdominal infection	0			
*C difficile*	0			
Total	31	6	5	18

a Some patients had multiple infections (4 of 35 cases did not meet criteria for an infection diagnosis).

b The following were included: *Enterobacter cloacae*, *Staphylococcus aureus*, and *E coli*.

c Vancomycin-resistant enterococci.

d *Klebsiella oxytoca*.

e Methicillin-resistant *Staphylococcus aureus*.

**Supplementary Table 9:** Outcomes for Individual Patients Who Triggered the Planned Composite End Point for the RCT

First component recorded	Day	Subsequent or concurrent component	Day	Subsequent or concurrent component	Day	Days in hospital, n	Days from composite endpoint to discharge, n	Alive at 30 days
Respiratory	13					13	0	Yes
Respiratory	7					23	16	Yes
Respiratory	15					16	1	Yes
Respiratory	8					23	15	No
Respiratory	3					12	9	Yes
Respiratory	9					17	8	Yes
Respiratory	4					6	2	Yes
Respiratory	3	Infection	9			15	12	Yes
Respiratory	3	Infection	5			28	25	Yes
Respiratory	3	Infection	6			17	14	Yes
Respiratory	3	Death	3			3	0	No
Respiratory	3	Infection	5	Circulation	9	23	20	Yes
Circulatory	6					15	9	Yes
Circulatory	9					21	12	Yes
Circulatory	3					3	0	Yes
Circulatory	3					6	3	Yes
Circulatory	3					15	12	Yes
Circulatory	9					14	5	Yes
Circulatory	4	Infection	8			32	28	Yes
Circulatory	11	Infection	15			27	16	Yes
Circulatory	10	Renal	12			14	8	Yes
Renal	3					11	8	Yes
Renal	3	Infection	4	Death	5	5	2	No
Renal	5	Infection + respiratory + circulatory	8	Cerebral	9	24	19	No
Infection	3					31	28	No
Infection	11					18	7	Yes
Infection	8					13	5	Yes
Infection	3					22	19	Yes
Infection	13					43	30	Yes
Infection	3					88	85	Yes
Infection	13	Respiratory	13			101	88	Yes
Infection	3	Respiratory	3			23	20	No
Infection	3	Circulatory	10			14	11	Yes
Infection	7	Renal	14	Death	15	15	8	No
Infection	3	Circulatory	5	Respiratory	6	34	31	Yes
Infection	3	Respiratory + circulatory	3	Renal	4	13	10	No
Infection	3	Respiratory + circulatory	3	Renal Death	5 10	10	7	No
Death	6					6	1	No

**Supplementary Table 10:** SAEs as Individually Reported

SAE description	Events, n	Causality determined by IDMC
New ascites	1	Unrelated
Renal impairment	1	Unrelated
Variceal bleeding (death)	3	Unrelated
Variceal bleeding (death)	1	Unlikely to be related
Pneumonia (death)	1	Unrelated
Death (decompensated cirrhosis)	4	Unrelated
Bronchogenic carcinoma and pleural effusion (death)	1	Unrelated

## Supplementary Materials and Methods

### Trial Management and Monitoring

#### Research steering group

The Research Steering Group operates on behalf of the funders to ensure that appropriate milestones have been met in the delivery of the trial. It consists of the chief investigator, an independent expert, and representatives of the Wellcome Trust and Department of Health.

#### Trial Management Group

The Trial Management Group comprises the chief investigator, Clinical Research Fellow, Clinical Project Manager, Trial Statistician, Trial Manager, Data Manager, Health Economist, and 5 trial site Principal Investigators. The Trial Management Group is responsible for developing the design, coordination, and strategic management of the trial.

#### Trial Steering Committee

The Trial Steering Committee is the independent group responsible for oversight of the trial to safeguard the interests of trial patients. The Trial Steering Committee provides advice to the chief investigator, Clinical Trials Unit, funder, and sponsor on all aspects of the trial through its independent chair.

#### Independent Data Monitoring Committee

The IDMC is responsible for safeguarding the interests of trial patients, monitoring the accumulating data, and making recommendations to the Trial Steering Committee on whether the trial should continue as planned. It comprises a clinical chair (independent hepatologist), independent gastroenterologist, and an independent statistician, all with expertise in Clinical Trials.

The participating hospitals and personnel were as follows: Dr G. Wright at Basildon University Hospital, 17 patients recruited; Dr J. Portal at Bristol Royal Infirmary, 14 patients recruited; Dr L. Corless at Hull Royal Infirmary, 11 patients recruited; Dr C. Lye Ch'ng at Singleton Hospital Swansea, 8 patients recruited; Dr P. Richardson at Royal Liverpool University Hospital, 7 patients recruited; Dr S. McPherson at Newcastle Freeman Hospital, 6 patients recruited; Dr Y. Kallis at Royal London Hospital, 6 patients recruited; Professor J. Metcalf at North Tees and Hartlepool, 6 patients recruited; Professor R. Jalan at Royal Free Hospital, 3 patients recruited; and Dr A. Elsharkawy at University Hospitals Birmingham, 2 patients recruited.
